# Noninvasive Terahertz Wave Binocular Stimulation Improves Cognition in Amyloid-β-Related Dementia

**DOI:** 10.34133/research.1268

**Published:** 2026-05-07

**Authors:** Hongwei Wu, Tingrong Zhang, Jing Ma, Junkai Yin, Jingzhou Liu, Hao Lin, Yun Yu, Yuanyuan He, Wuzhong Cheng, Zihua Song, Chao Chang

**Affiliations:** ^1^ Innovation Laboratory of Terahertz Biophysics, National Innovation Institute of Defense Technology, Beijing 100071, China.; ^2^ School of Life Science and Technology, Xi’an Jiaotong University, Xi’an 710049, China.; ^3^ School of Safety Engineering, University of Emergency Management, Hebei 065201, China.; ^4^ Capital Medical University, Beijing 100069, China.; ^5^ School of Physics, Peking University, Beijing 100081, China.

## Abstract

Terahertz (THz) waves occupy a unique spectral region where they can resonantly interact with collective vibrational modes of biomolecules, thereby modulating protein conformation and subsequently affecting neural signaling. However, whether noninvasive THz wave stimulation can be extended to Alzheimer’s disease and related dementias (ADRD) remains unknown. Here, we demonstrate that 14 d of noninvasive THz wave binocular stimulation (33 THz) effectively restores cognitive performance and neural homeostasis in Aβ_1–42_-induced dementia mice, a widely used experimental model of ADRD with the features including neuroinflammation and cognitive decline. Behavioral assessments revealed a marked restoration of spatial learning and memory ability, with a 53.1% ± 22.0% improvement compared with untreated dementia mice. Furthermore, THz wave stimulation reduced sleep fragmentation and restored physiological sleep–wake dynamics, indicating improved neural homeostasis. In the hippocampus, binocular stimulation attenuated microglial activation and normalized the levels of pro-inflammatory cytokines including interleukin-1β and tumor necrosis factor-α. Concurrently, THz wave stimulation activated hippocampal cyclic adenosine monophosphate (cAMP)–cAMP-response element binding protein (CREB)–brain-derived neurotrophic factor (BDNF) signaling, promoting neurotrophic support and re-establishing cholinergic functional integrity. Our findings demonstrate that THz wave binocular stimulation is a noninvasive approach capable of improving cognitive function in a dementia mouse model, offering a potential strategy to restore brain function in neurodegenerative diseases.

## Introduction

Alzheimer’s disease and related dementias (ADRD) represent one of the greatest unmet challenges in modern medicine [1]. Characterized by memory loss and progressive cognitive decline due to neurodegeneration, ADRD currently affect more than 55 million people worldwide, imposing immense emotional and economic burdens on patients, families, and healthcare systems [2]. Among these disorders, Alzheimer’s disease (AD) remains the predominant form, pathologically defined by amyloid-β (Aβ) deposition, tau aggregation, and chronic neuroinflammation [3]. Traditional pharmacological treatments have shown limited efficacy [4,5], and this ongoing therapeutic impasse emphasizes the critical need for innovative approaches to not only reverse cognitive decline but also restore neural function in dementia.

Noninvasive modulation of brain activity is a central goal in neuroscience research and clinical practice. Recent studies have demonstrated that gamma-frequency (~40 Hz) oscillations driven by light or sound can directly modulate brain activity [6,7]. These oscillations have been shown to ameliorate AD-related pathology in animal models [8,9], including reducing Aβ deposition while partially improving cognitive performance [10–12]. A host of alternative neuromodulation strategies, such as electrical and magnetic stimulation [13,14], as well as near-infrared photo-biomodulation [15,16], have also been reported to enhance neuronal excitability, mitochondrial metabolism, and cerebral perfusion, further supporting the feasibility of noninvasive regulation of brain function.

Within this emerging framework, terahertz (THz) radiation, which bridges the gap between infrared and microwave frequencies, represents a particularly intriguing modality. THz waves can resonate with collective molecular vibrations and hydrogen-bond dynamics in biomolecules, enabling direct influence on neuronal processes [17]. Experimental studies suggest that THz wave exposure promotes neuronal growth [18,19] and neuro single transduction [20–22], enhances DNA unwinding and synthesis [23], modulates protein conformation [24–27], improves behavioral and sensory performance in animal models [28–31], and even serves as a new energy source for biological activity [32]. Together, these findings point to THz wave neuromodulation as a promising and largely untapped strategy for restoring neural and cognitive function in dementia.

In this study, we established a dementia mouse model through intracerebroventricular injection of Aβ_1–42_ [33], a widely recognized AD modeling method that recapitulates its cognitive and neuropathological deficits. Using this model, we investigated the therapeutic potential of THz wave binocular stimulation as a noninvasive neuromodulation strategy, different from any other THz wave stimulation method reported before (Table S1). We found that our stimulation paradigm restored spatial learning and memory performance, normalized sleep architecture, and promoted neural homeostasis. Furthermore, it enhanced cyclic adenosine monophosphate (cAMP) signaling and brain-derived neurotrophic factor (BDNF)-dependent cholinergic activity, while suppressing pro-inflammatory cytokines, including interleukin-1β (IL-1β) and tumor necrosis factor-α (TNF-α), to near-normal levels. Together, these findings demonstrate that THz wave binocular stimulation is a noninvasive approach capable of restoring cognitive performance in our dementia mouse model.

## Results

### Establishment of the dementia mouse model

**Fig. 1.**
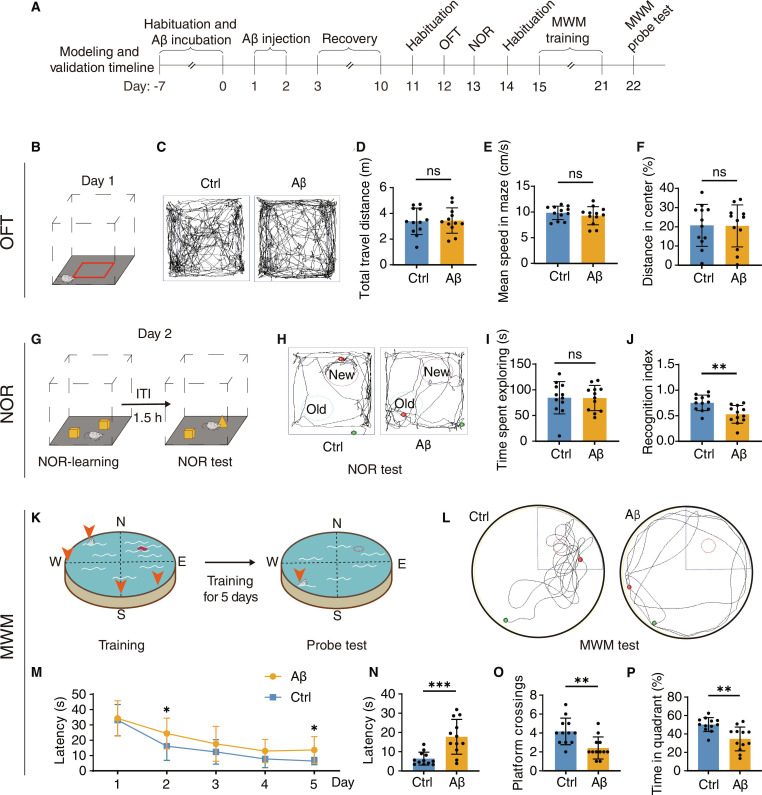
Intraventricular injection of Aβ_1–42_-induced dementia model with cognitive impairment. (A) Timeline of model establishment. (B) Schematic diagram of the open-field test (OFT). (C) Comparison of movement trajectories in the OFT between the Aβ model and control groups. (D) Mean speed (cm/s) during novel object recognition (NOR) test (*n* = 12 per group). (E) Distance traveled in the center area during the OFT (*n* = 12 per group). (F) Total distance (cm) traveled during the OFT (*n* = 12 per group). (G) Schematic diagram of the NOR test. (H) Comparison of movement trajectories in NOR between the Aβ model and control groups. (I) Time spent exploring both objects in learning phrase (*n* = 12 per group). (J) Recognition index of NOR test (*n* = 12 per group). (K) Schematic diagram of the Morris water maze (MWM). (L) Comparison of movement trajectories of test phrase in the MWM between the Aβ model and control groups. (M) Escape latencies (s) change of Aβ model mice and ctrl group in the MWM (*n* = 12 per group). (N) Escape latencies (s) during the probe trial (*n* = 12 per group). (O) Number of platforms crossing during the probe trial (*n* = 12 per group). (P) Time (s) spent swimming in the goal quadrant during the probe test (*n* = 12 per group). Data are shown as means ± SEM.*P < 0.05; **P < 0.01; ***P <0.001; ns, not significant. Two-tailed unpaired Student *t* test was used for (D), (E), (F), (I), (J), (M), (N), (O), and (P).

In line with the timeline described in Fig. 1A, we first establish the dementia mouse model by injecting Aβ_1–42_ oligomers bilaterally into the lateral ventricles of Kunming (KM) mice and verify its reliability through behavioral test 1 week after infection, confirming the presence of typical dementia-like symptoms. In the open-field test (OFT) (Fig. 1B and C), no marked differences were observed between groups in total distance traveled (Fig. 1D) or average velocity (Fig. 1E), indicating comparable locomotor capacity. The proportion of distance traveled within the central zone was also similar (Fig. 1F), indicating that neither the surgical procedure nor the Aβ injection induced anxiety-like behaviors. In the novel object recognition (NOR) test (Fig. 1G), which relies on the innate tendency of rodents to explore novel stimuli, Aβ-injected mice (Aβ) exhibited the same exploratory behavior as mice injected with phosphate-buffered saline (PBS) (Ctrl), but a significantly decreased discrimination index (Fig. 1H to J). The control group demonstrated a clear preference for exploring the novel object, while the Aβ group exhibited an inability to differentiate between familiar and novel objects, suggesting impaired recognition memory despite preserved exploratory drive.

Spatial learning and memory were further evaluated using the Morris water maze (MWM) (Fig. 1K and L). During the acquisition phase, the Aβ group exhibited significantly prolonged escape latencies compared with the control group (Fig. 1M). In the probe trial, mice in the Aβ group exhibited a significant reduction in the times they crossed the former platform location and a decrease in the duration of time spent in the target quadrant, demonstrating profound spatial memory deficits when compared with the control group (Fig. 1N to P). The collective analysis of behavioral results establishes a robust model of dementia characterized by impaired cognitive function and intact motor ability, consistent with the clinical features of AD.

In addition to behavioral deficits, molecular analyses further confirmed the validity of the Aβ_1–42_-induced dementia model: enzyme-linked immunosorbent assays (ELISAs) revealed a time-dependent decline in BDNF concentration within both the hippocampus (HPC) and basal forebrain (BF) following Aβ injection (Fig. S1A), indicating a progressive loss of neurotrophic support during disease development. Western blot analysis further showed increased expression of the astrocytic marker glial fibrillary acidic protein (GFAP) and decreased levels of the neuronal cytoskeletal protein NF-L in both regions (Fig. S1B), consistent with astrocyte activation and neuronal injury [34].

Together, these findings corroborate that intracerebroventricular Aβ_1–42_ injection induces neuroinflammatory and degenerative alterations that accompany the observed cognitive impairments, thereby establishing a reliable model of dementia.

### Development and safety evaluation of the THz wave binocular stimulation device

**Fig. 2.**
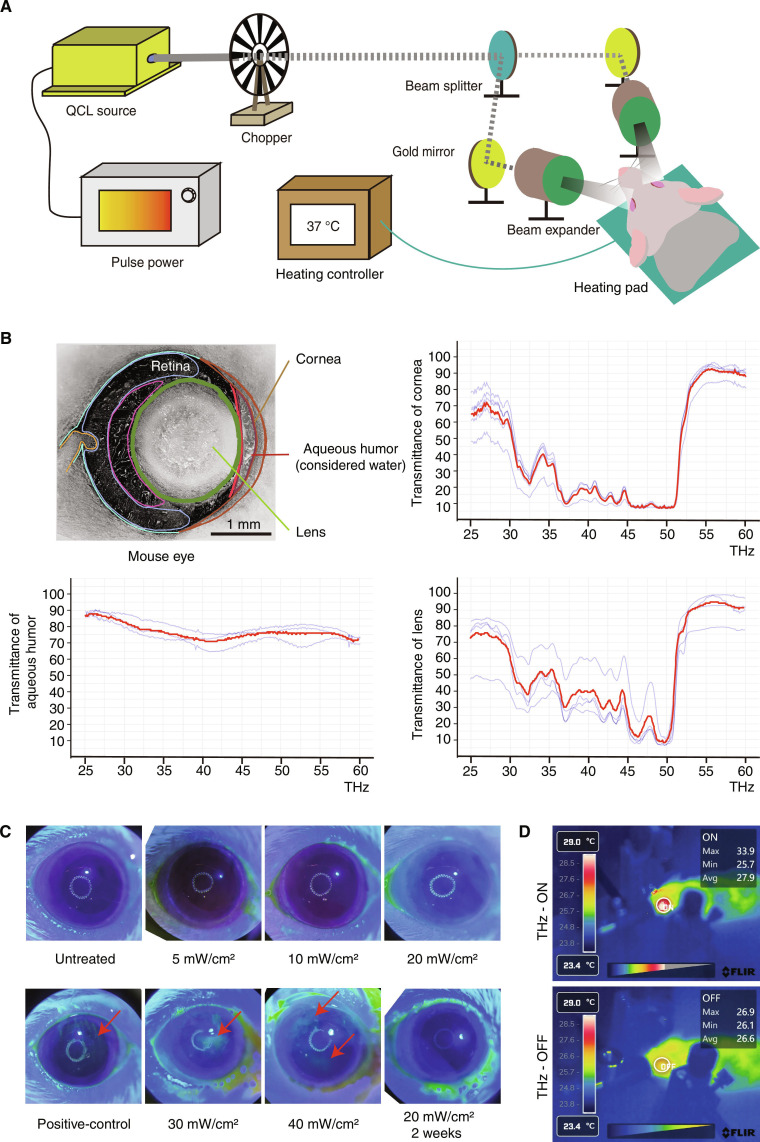
Setup of terahertz (THz) wave binocular stimulation platform and exploration of stimulation conditions. (A) Schematic diagram of the THz wave optical path, including the QCL source, optical chopper, lens group, body temperature maintenance blanket, and anesthesia machine. (B) Schematic diagram of mouse eye anatomy and transmittance analysis of main ocular parts. (C) Detection of THz laser-induced corneal damage at different power densities using sodium fluorescein (yellow areas represent damaged regions, with needle scratching used as a positive control). (D) Temperature safety monitoring during stimulation at selected power density (20 mW/cm^2^).

To investigate the therapeutic potential of THz wave binocular stimulation, we constructed a custom optical setup delivering 33 THz flickering light at 40 Hz. The stimulation pathway was aligned to target the ocular region bilaterally (Fig. 2A). Considering that biological tissues exhibit intrinsic absorption in the THz range [35], we measured the transmission spectra of major ocular structures using a Fourier-transform infrared spectrometer, revealing that 33 THz wave exhibited sufficient transmissivity across the cornea and crystalline lens to reach the posterior ocular compartment (Fig. 2B). However, consistent with previous reports on skull transmittance at THz frequencies, 33 THz radiation shows limited penetration through cranial bone [36], indicating that the effective irradiation range at this frequency is confined to the ocular region rather than direct bulk-brain exposure.

To further verify that THz irradiation effectively stimulates the intended ocular target, we additionally performed c-Fos immunofluorescence analysis in ocular tissues following THz wave binocular stimulation (Fig. S4A), which revealed a significant increase in c-Fos expression compared with nonirradiated controls, confirming local biological activation at the eye. These observations support the notion that THz wave stimulation primarily engages ocular pathways, rather than directly penetrating the skull to act on deeper brain structures.

To assess the biosafety of 33 THz wave binocular stimulation, fluorescein sodium staining was performed on the cornea following 40 min of stimulation at different power densities (5, 10, 20, 30, and 40 mW/cm^2^), using positive controls generated by needle tip as references. No corneal structural or vascular damage was detected below 20 mW/cm^2^ (Fig. 2C), then selected as the optimal stimulation intensity. The local temperature increases in the irradiated area remained within 1.3 ± 2.3 °C (Fig. 2D), confirming the thermal safety of the stimulation process.

### THz wave binocular stimulation restores cognitive function in dementia mice

**Fig. 3.**
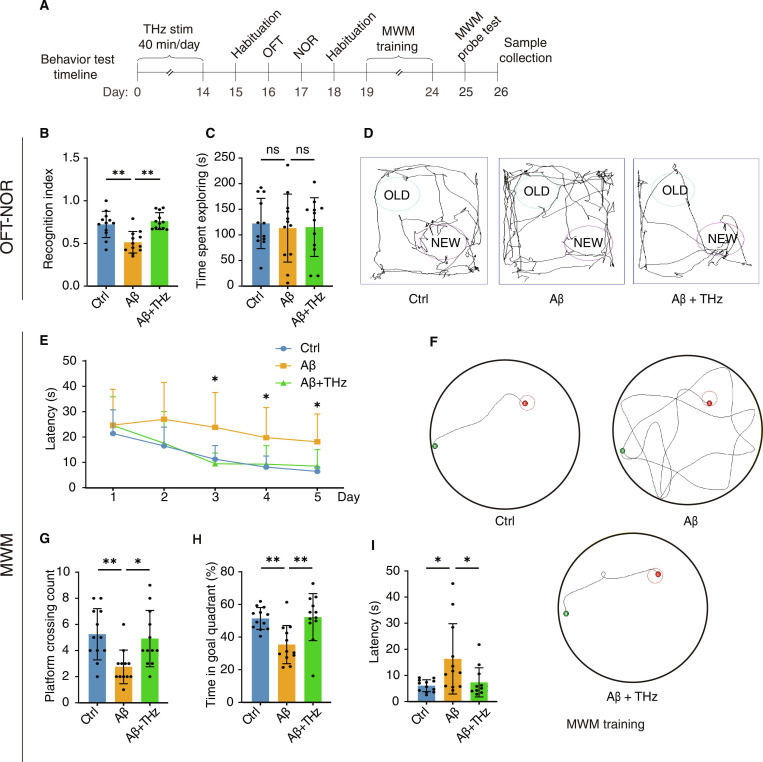
THz wave binocular stimulation restores cognitive function in dementia model mice. (A) Timeline of THz wave binocular stimulation and subsequent behavioral tests. (B) Recognition index of NOR test (*n* = 12 per group). (C) Time spent exploring both objects in learning phrase (*n* = 12 per group). (D) Comparison of movement trajectories of testing phrase in NOR test between 3 groups. (E) Progression of escape latencies (s) for the 3 groups across training days in the Morris water maze (*n* = 12 per group). (F) Comparison of movement trajectories of training phrase in the Morris water maze between 3 groups. (G) Number of platforms crossing during the probe trial (*n* = 12 per group). (H) Time (s) spent swimming in the goal quadrant during the probe test (*n* = 12 per group). (I) Escape latencies (s) during the probe trial (*n* = 12 per group). Data are shown as means ± SEM.*P < 0.05; **P < 0.01; ns, not significant. One-way analysis of variance (ANOVA) was used for (B), (C), (D), (E), (G), (H), and (I).

Inspired by the findings of noninvasive THz wave therapy, we investigated whether binocular exposure to a 33-THz wave at 40 Hz may restore cognitive function in mice with dementia (Fig. 3A). We use OFT to assess emotional state after stimulation and found that neither Aβ injection nor stimulation altered basal locomotion or anxiety-like behavior (Fig. S2A), thus excluding motor or motivational confounds for subsequent cognitive measures. Notably, binocular THz wave stimulation robustly rescued recognition memory of the Aβ group in the NOR task (Fig. 3B), but no differences in exploration desire were detected among the mice in the control, Aβ, and Aβ + THz groups (Fig. 3C). The stimulation increased the recognition index by 49% compared to Aβ alone, indicating that object recognition was restored while the exploratory drive remained intact (Fig. 3D).

On the final training day of the MWM, the Aβ group prolonged latency by 184% compared to the control group, while the Aβ + THz group reduced latency by 53.1% ± 22.0% compared to the Aβ group, approaching the values of the control group (Fig. 3E and F). In the probe trial (Fig. 3G), the Aβ group exhibited a 48% reduction in platform crossings relative to the control group, while the Aβ + THz group demonstrated a 79% increase in crossings compared to the Aβ group and remained within 6% of the control group, indicating near-complete recovery of search precision. The Aβ group produced 31% reduction in time spent at the target quadrant compared with the control group, while the Aβ + THz group showed an increase in target time of 47% relative to the Aβ group and slightly exceeded the control group by ~1.6% (Fig. 3H), consistent with full normalization of spatial memory allocation. Finally, the Aβ group exhibited a 168% increase in the latency to first enter the former platform zone relative to the control group, whereas the Aβ + THz group demonstrated a 55% reduction in this parameter compared to the Aβ group, leaving a modest 21% residual difference from control within the observed variance (Fig. 3I). A comprehensive analysis of mice that underwent the MWM both before and after stimulation (with platform locations changed) revealed that THz-illuminated mice exhibited significant improvements in all measured parameters, whereas the nonilluminated group was not affected (Fig. S2B). Together, these data demonstrate that a 2-week course of THz wave binocular stimulation restores recognition and spatial memory in Aβ-induced dementia mice to control levels.

### THz wave binocular stimulation promotes sleep in dementia mice

**Fig. 4.**
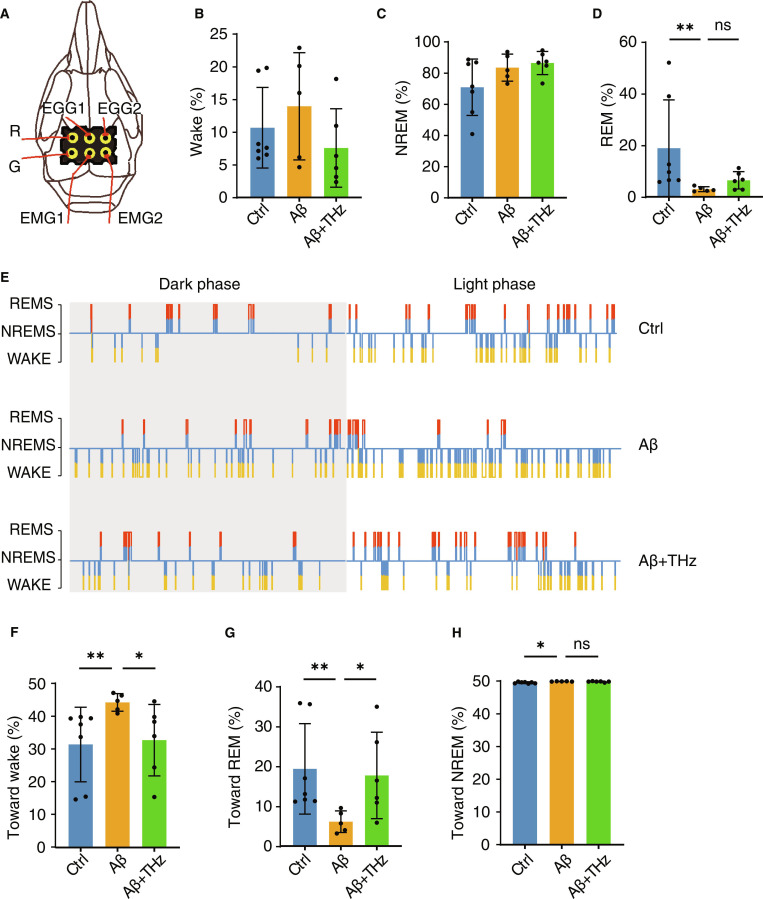
THz wave binocular stimulation ameliorates Aβ-induced sleep architecture abnormalities. (A) Location of the electrode socket on the mouse skull. (B to D) Percentage of time spent in Wake, non-rapid eye movement sleep (NREM), and rapid eye movement sleep (REM) states over a 24-h period (*n* = 5 to 7 per group). (E) Schematic representation of state transition analysis across Wake, NREM, and REM over a 24-h period. (F to H) Proportion of transitions toward Wake, NREM, and REM states over a 24-h period (*n* = 5 to 7 per group). Data are shown as means ± SEM.*P < 0.05; **P < 0.01; ns, not significant. Statistical analysis was performed using the Kruskal–Wallis test followed by Dunn’s multiple-comparison test for (B), (C), (D), (F), (G), and (H).

Given that the 33-THz wave can restore cognitive function in Aβ_1–42_-induced dementia mice, we further assessed neural network-level activity using electroencephalography (EEG) combined with electromyography recordings (Fig. 4A). Following a 24-h period of uninterrupted signal recording, the signals were categorized into 3 states: wakefulness (Wake), non-rapid eye movement sleep (NREM), and rapid eye movement sleep (REM). The results showed that, compared with the control group, there were no marked differences in total Wake or NREM duration in the Aβ group, whereas time spent in REM was markedly reduced (Fig. 4B to D), indicating that Aβ injection may preferentially disrupt REM sleep architecture. Notably, THz wave binocular stimulation did not significantly restore the reduction in REM sleep. Considering the potential influence of circadian rhythms on sleep architecture, we further analyzed the distribution of sleep states during light and dark phases. During the light phase, there was a slight increase in REM sleep time, although this did not reach statistical significance (Fig. S3A). Similarly, analysis of the overall power spectrum and individual frequency bands (δ, θ, α, β, and γ) revealed no marked differences among the groups (Fig. S3B and C).

Sleep fragmentation is a common feature in AD patients [37]; we quantified the proportion of state transitions among Wake, NREM, and REM states over a continuous 24-h recording period (Fig. 4E). In the Aβ group, the proportion of Wake-associated transitions was significantly elevated, and this was partially alleviated following binocular stimulation (Fig. 4F). This abnormal increase in Wake transitions was attributable to a pronounced reduction in NREM-to-REM transitions, rather than others toward NREM (Fig. 4G and H). When considering circadian phase, the changes in transition frequency were most evident during the light phase, with minor alterations observed during the dark phase (Fig. S3D). Collectively, these findings indicated that THz wave binocular stimulation effectively ameliorated the abnormal transition frequency in sleep architecture, suggesting that this intervention may alleviate Aβ-induced sleep fragmentation, thereby enhancing the stability and flexibility of neural network activity and ultimately contributing to the recovery of cognitive function.

### Proteomic profiling revealed changes in cognition-related protein and pathways within the HPC following THz wave binocular stimulation

To search the specific brain regions involved in the THz wave binocular stimulation, c-Fos immunostaining was performed in healthy mice following a single stimulation session. c-Fos expression was detected in multiple areas across the BF–HPC axis, which are critically involved in autonomic regulation and memory processing. Quantifications revealed a significant increase of c-Fos^+^ cells induced by the binocular stimulation (Fig. S4A to D), suggesting direct or indirect functional connectivity from the retina to the dentate gyrus of the HPC and BF.

**Fig. 5.**
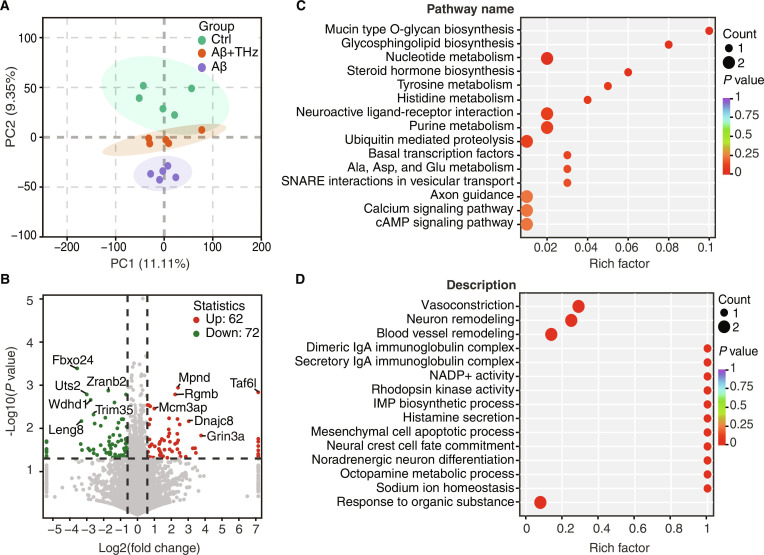
Proteomic analysis reveals changes in proteins and pathways within the HPC following THz wave binocular stimulation. (A) Principal component analysis (PCA) of the proteomic analyses across 3 experimental groups (*n* = 5 per group). (B) Volcano plot of differential protein expression compared Aβ + THz with Aβ (*n* = 5 per group). (C) Kyoto Encyclopedia of Genes and Genomes pathway enrichment analysis of proteins significantly up-regulated compared Aβ + THz with Aβ (*n* = 5 per group). (D) Gene Ontology enrichment analysis (biological process) for up-regulated proteins compared Aβ + THz with Aβ (*n* = 5 per group).

Considering the c-Fos activation and the well-established role of the HPC in memory formation, we conducted a proteomic analysis of this area to further substantiate the effects of THz wave binocular stimulation on the HPC. Principal component analysis revealed a clear separation between the THz-treated and control (Aβ) groups, indicating a robust remodeling of the hippocampal function (Fig. 5A). The volcano plot consistently identified 134 differentially expressed proteins (62 up-regulated and 72 down-regulated) in the Aβ + THz group compared to the Aβ group (Fig. 5B). Among the enriched Kyoto Encyclopedia of Genes and Genomes pathways (Fig. 5C), 2 categories were particularly consistent with the restoration of hippocampal function. Calcium signaling, which plays a key role in regulating synaptic transmission, growth cone dynamics, and activity-dependent plasticity, was markedly up-regulated in the Aβ + THz group, suggesting enhanced neuronal excitability and plastic remodeling. Concurrently, axon guidance pathways were activated, indicating that binocular stimulation may facilitate structural rewiring of damaged circuits by promoting axonal navigation and synaptic re-establishment. Further Gene Ontology enrichment analysis revealed a significant increase in the proteins linked to neuron remodeling following binocular stimulation (Fig. 5D). Additionally, the up-regulation of proteins associated with sodium ion homeostasis points to improved ionic balance and neuronal responsiveness, which are essential for restoring stable network activity. Together, these pathway-level signatures align with our functional findings and suggest plastic changes at the protein level taking place within the HPC after THz wave binocular stimulation.

### THz wave binocular stimulation affects cAMP–CREB–BDNF signaling and suppresses neuroinflammation in the HPC

**Fig. 6.**
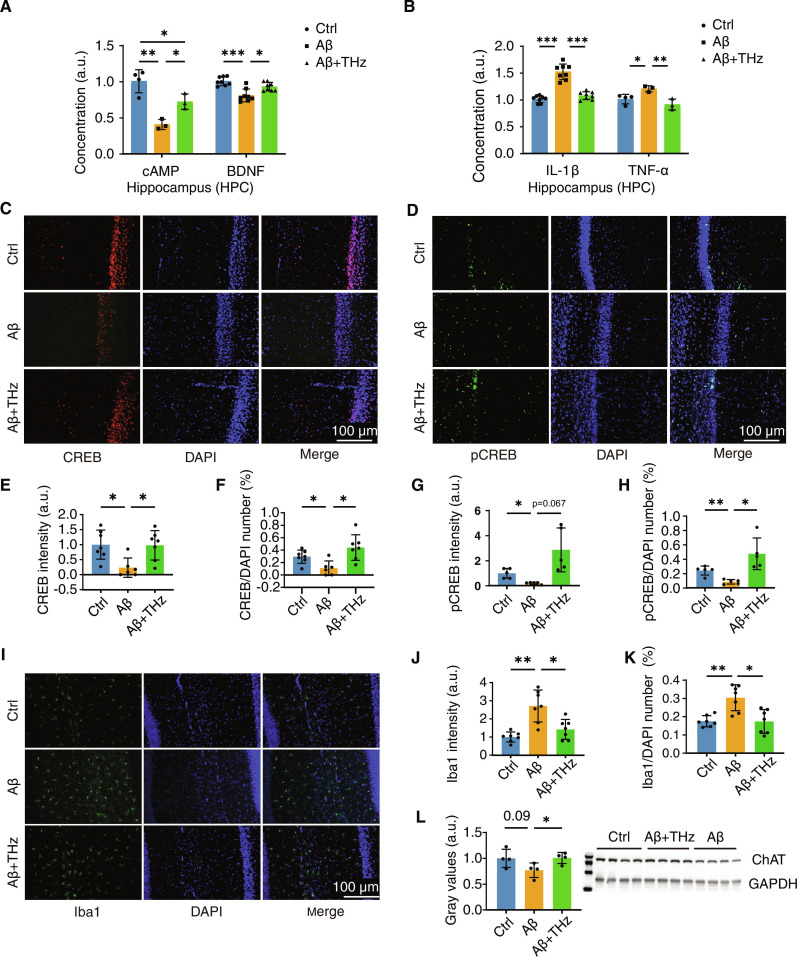
THz wave binocular stimulation restores the cAMP (cyclic adenosine monophosphate)–CREB (cAMP-response element binding protein)–BDNF (brain-derived neurotrophic factor)–ChAT (choline acetyltransferase) signaling and suppresses neuroinflammation in the HPC. (A) Relative cAMP and BDNF levels in HPC in dementia mice with/without binocular stimulation for 2 weeks (normalized to control, *n* = 3 to 8 per group). (B) Relative IL-1β and TNF-α levels in HPC of dementia mice with/without binocular stimulation for 2 weeks (normalized to control, *n* = 3 to 8 per group). (C) Immunofluorescence staining for CREB (red) in dementia mice’s HPC with/without binocular stimulation for 2 weeks (*n* = 6 to 7 per group, scale bar: 100 μm). (D) Immunofluorescence staining for pCREB (green) in dementia mice’s HPC with/without binocular stimulation for 2 weeks (*n* = 5 per group, scale bar: 100 μm). (E) Relative CREB-positive plaque intensity in HPC (*n* = 6 to 7 per group). (F) Average number ratio of CREB-positive cells in HPC (*n* = 6 to 7 per group). (G) Relative pCREB-positive plaque intensity in HPC (*n* = 5 per group). (H) Average number ratio of pCREB-positive cells in HPC (*n* = 5 per group). (I) Immunofluorescence staining for Iba1 (green) in dementia mice’s HPC with/without binocular stimulation for 2 weeks (*n* = 7 per group, scale bar: 100 μm). (J) Relative Iba1-positive plaque intensity in HPC (*n* = 7 per group). (K) Average number ratio of Iba1-positive cells in HPC (*n* = 7 per group). (L) Relative gray values of ChAT in HPC of dementia mice with/without binocular stimulation for 2 weeks (*n* = 4 per group, normalized to control). Data are shown as means ± SEM. *P< 0.05, **P< 0.01, ***P< 0.001; ns, not significant. One-way ANOVA was used for (A), (B), (E), (F), (G), (H), (J), (K), and (L).

To further explore potential molecular mechanisms through which cognitive restoration was stimulated by THz, we explored the expression of a number of molecules previously reported to be involved in the cognitive function [38]. It is widely acknowledged that cAMP signaling activates the transcription factor cAMP response element-binding protein (CREB), which in turn promotes BDNF synthesis and release [39]. ELISA measurements revealed that hippocampal cAMP concentrations were significantly reduced in Aβ mice and partially restored by binocular stimulation, corresponding to a 75% loss in Aβ and 43% recovery after THz treatment (Fig. 6A). Concurrently, a decline in hippocampal BDNF levels was observed in Aβ mice, with THz wave exposure eliciting an improvement (15% reduction vs. control; 28% gain vs. Aβ). Immunofluorescence analysis further demonstrated restoration of CREB and phosphorylated CREB (pCREB) activity in the Aβ + THz group (Fig. 6C to H). These findings indicate that THz wave binocular stimulation reactivates the cAMP–CREB transcriptional cascade, which was otherwise suppressed in the Aβ model.

BDNF has been shown to regulate microglial activity [40], which becomes aberrantly activated following Aβ injection into the brain [41–43] and plays an essential role in maintaining neural homeostasis [44,45]. To investigate whether hippocampal microglia are involved in binocular stimulation, Iba1 (a recognized marker for microglia) immunostaining showed that both the Iba1 intensity and number were significantly increased in the Aβ group compared to the control group, and returned to normal following stimulation (Fig. 6I to K). Consistently, ELISA quantification of cytokines revealed that hippocampal IL-1β and TNF-α were markedly elevated in Aβ mice but restored to near-normal levels by binocular stimulation, while no changes were observed in IL-6 or IL-10 (Fig. 6B and Fig. S5A). In the BF, THz wave treatment significantly normalized all 4 cytokines tested (IL-1β, TNF-α, IL-6, and IL-10) (Fig. S5B) and also observed the change of cAMP concentration (Fig. S5C). Given that BDNF is known to promote the survival and function of cholinergic neurons [46], we examined ChAT protein levels by Western blot: levels declined from 1.00 ± 0.18 in control mice to 0.77 ± 0.14 in Aβ mice, but were restored to 1.01 ± 0.11 following THz wave stimulation (Fig. 6L).

Collectively, these results demonstrate that THz wave binocular stimulation correlates with the coordinated normalization of cAMP–CREB–BDNF–ChAT signaling and microglial activity, which we interpret as downstream molecular and cellular events underlying functional recovery. The up-regulation of BDNF, coupled with the restoration of microglial homeostatic phenotypes, represents enhanced neuronal support and the resolution of neuroinflammatory stress in Aβ-injected mice. These molecular and cellular alterations align with the observed recovery of cognitive function and sleep–wake behavior, suggesting that THz wave binocular stimulation fosters neuroimmune homeostasis and adaptive plasticity via indirect and integrative mechanisms.

## Discussion

This study demonstrates that Aβ_1–42_ injection induces cognitive and sleep-related impairments in KM mice, both of which are effectively reversed by binocular THz stimulation. These effects are associated with the coordinated regulation of hippocampal cAMP–CREB–BDNF signaling and microglial activation, which collectively contribute to the restoration of cognition-related behaviors.

Previous studies have demonstrated that rhythmic 40-Hz sensory stimulation can entrain gamma oscillations and mitigate Alzheimer’s-like pathology [10,11]. However, under comparable stimulation conditions, 40-Hz white light flicker failed to restore behavioral performance in our dementia model mice. In contrast, unmodulated THz wave stimulation effectively rescued cognitive deficits, while 40-Hz-modulated THz wave stimulation further improved the stability of these effects (Fig. S6A to I). These findings indicate that the therapeutic benefit observed here cannot be solely explained by classical gamma entrainment. Unlike conventional sensory flicker paradigms, THz wave stimulation may influence neural systems in a state-independent manner, potentially through biophysical interactions at the retinal and molecular levels that extend beyond cortical rhythm synchronization.

While the present study establishes the therapeutic efficacy of THz wave binocular stimulation and identifies associated neuroinflammatory and regional changes, the underlying mechanisms are likely to involve multilevel neural and immunological processes along the eye–brain axis. Given the ability of THz photons to stimulate the retina and potentially access central circuits via the optic nerve, together with the shared immune environment between the eye and the brain [47], the neuromodulatory effects observed here may extend beyond classical gamma entrainment. Rather than acting solely through cortical rhythm synchronization, THz wave stimulation may engage coordinated retinal, neural, and immune interfaces that collectively contribute to state-independent neuroprotection.

It should also be noted that stimulation in the present study was conducted under anesthesia due to current optical system constraints. Although appropriate controls were implemented to minimize confounding effects, future studies in awake, freely behaving conditions will be essential to fully characterize physiological responsiveness and translational potential. In parallel, recent advances in THz wave engineering, including dynamically tunable metasurfaces [48] and compact modulation platforms [49,50], may enable more precise, scalable, and noninvasive delivery paradigms, further facilitating mechanistic investigation and clinical adaptation of THz wave neuromodulation.

Collectively, our findings establish THz wave binocular stimulation as a previously unexplored modality of optical neuromodulation capable of coordinating neural and immune homeostasis. By extending noninvasive electromagnetic neuromodulation into the THz spectral domain, this work broadens both the conceptual and physical boundaries of light-based brain modulation. These results provide a foundational framework for the developing state-independent, noninvasive strategies to restore brain function in neurodegenerative disorders.

## Methods

### Experimental model and animal details

#### Mice

KM mice (8 weeks old, purchased from SPF Biotech) were housed in a specific pathogen-free facility under a standard 12-h light/12-h dark cycle. To maintain optimal living conditions, average housing density was 4 mice per cage. Food and water were provided without limit. When additional experimental groups were introduced, an equal number of control groups were included accordingly to exclude the effects of anesthesia, ambient temperature, and other laboratory stimuli on the experimental results. After grouping or experimentation, all mice remained in their original cages, labeled as the home cage. To minimize animal stress, all animals were acclimated for 1 week before the experiment and received daily 5-min handling sessions. All animal procedures were approved by the Institutional Animal Care and Use Committee of the Academy of Military Medical Sciences.

#### Aβ_1–42_ oligomer preparation

Aβ_1–42_ peptides (1 mg, 03-112, Thermo Fisher) were dissolved in 5 μl of dimethyl sulfoxide to create a stock solution. The solution was then diluted by adding 245 μl of PBS (G4202, Servicebio) to achieve a final volume of 250 μl (4 μg/μl). The diluted solution was incubated at 37 °C for 5 d to allow oligomerization. After incubation, the oligomers were used for subsequent experiments.

#### Modeling surgery

We established the dementia mouse model by injecting Aβ oligomers into the lateral ventricles [51–53]. Aβ oligomers or PBS were injected in mice brain by intracerebroventricular injection using custom-made glass electrode injector. Mice were anesthetized with 1.25% 2,2,2-tribromoethanol (T708333, Macklin) based on their body weight and then their scalp hair was carefully shaved; surgery area was disinfected with iodine tincture to prevent infection. The animals were then placed in a stereotaxic frame, and ophthalmic ointment (erythromycin) was applied to protect the eyes from potential injury. After placement, the scalp was cut and then a small incision was made on the skull to expose the brain. The injection coordinates were as follows: *X* (M/L): ±1 mm, *Y* (A/P): −0.22 mm, *Z* (D/V): −2.43 mm relative to bregma. A total volume of 2 μl of Aβ oligomer solution was injected into each lateral ventricle at a speed of 500 nl per minute. The needle was left in place for 15 min post-injection to allow for diffusion of the solution, and was then slowly withdrawn. After the procedure, the wound was sutured, and the mice were allowed to recover for 1 week before further experiment.

### Method details

#### Noninvasive THz wave binocular stimulation system

The THz wave binocular stimulation system employed a quantum cascade laser (QCL) with a center frequency 33 THz wave as the radiation source. The laser was powered by a pulsed current supply (8 to 15 V, 300 to 800 mA). To achieve frequency modulation and bilateral stimulation, the THz beam emitted from the QCL was modulated using an optical chopper (set to 40 Hz, 12.5 ms on–off) and subsequently divided into 2 beams by a beam splitter (Thorlabs). Each beam was directed by a reflection mirror (Thorlabs) into a custom-built Keplerian beam expander (Edmund Optics) for power density adjustment and beam collimation. The adjusted THz beams were then directed toward the ocular area of the mouse at controlled power densities. The entire platform was placed on an optical platform with blackout curtains to ensure a dark environment during irradiation, effectively preventing interference from external light sources.

During stimulation, mice were lightly anesthetized with low-concentration isoflurane (0.8% to 1.5%), and their body temperature was maintained at physiological levels using a temperature-controlled heating pad, while stimulation at a safe power density of 20 mW/cm^2^ was applied for 40 min daily over a continuous 14-day period. The mice were divided into 3 groups: control (ctrl), Aβ-injected (Aβ), and Aβ-injected followed by THz wave binocular stimulation (Aβ + THz). To exclude the effects of factors other than THz wave binocular stimulation including anesthesia, all mice were individually placed on the stimulation platform, with the only difference being whether the light source was turned on, ensuring that any potential confounding effects from the platform environment, anesthesia, or ambient conditions were controlled.

#### Measurement of light power density (90/10 knife-edge method)

Since the power density on the cross-section of the THz laser emitted from the source follows a Gaussian distribution, the Gaussian beam radius was used to quantify the power density for more accurate irradiation dose calculation. Beam size and on-target power density were quantified using the 90/10 knife-edge method [54]. The THz beam was directed to the measurement plane conjugate to the mouse eye via the experimental beam path. A detection card mounted on a manual micrometer translation stage was positioned perpendicular to the propagation axis and translated laterally across the beam. First, the power of THz beam (P) was measured without occlusion using thermopile power meter (Orphi). Next, the 90% and 10% power levels ($P_{90}$/$P_{10}$) were calculated, and the detection card was translated until the power matched the $P_{90}$/$P_{10}$ power levels. The position at these points was recorded as 𝑥_90_ and 𝑥_10_, and the difference was denoted as Δ𝑥 = 𝑥_90_ − 𝑥_10_. Assuming a Gaussian intensity distribution, the 1/e^2^ beam radius (𝑤) was then calculated using the empirical relation:

$$w=\frac{\Delta x}{1.28}$$ (1)

Assuming a circular beam spot, the power density (I) was calculated as:

$$I=\frac{P}{{\pi w}^{2}}$$ (2)

Final stimulation for in vivo experiments was adjusted to 20 mW/cm^2^ at the ocular area, verified by thermal imaging camera prior to animal exposure.

#### Sample collection

For brain region samples used for homogenization, KM mice were anesthetized with tribromoethanol and euthanized by decapitation. The HPC and BF were rapidly dissected, placed into cryovials, and then frozen in liquid nitrogen (samples can be stored short term at −80 °C).

For whole brain samples used for sectioning, KM mice were anesthetized with tribromoethanol and perfusion with 4% paraformaldehyde (PFA, G1101, Servicebio) to achieve systemic fixation. Following perfusion, the animals were decapitated, and the brain was carefully removed and post-fixed in PFA at 4 °C overnight. After fixation, the brain was subjected to a sucrose (V900116, Sigma-Aldrich) gradient (10%, 20%, and 30%) for dehydration. Upon completion, the brains were embedded in optimum cutting temperature (OCT) compound (HBDY-4985, SAKURA) and rapidly frozen for sectioning (samples can be stored short term at −80 °C).

#### Western blotting

Frozen samples were homogenized in RIPA buffer (50 mM Tris-HCl, pH 8.0, 150 mM NaCl, 1% NP-40, 0.5% sodium deoxycholate, and 0.1% sodium dodecyl sulfate) with phosphatase and protease inhibitors (05892791001, 04906837001, Roche) based on tissue weight (1 mg:10 μl) using a homogenizer (JINGXIN), incubated on ice for 10 min, and rotated at 4 °C for 40 min. The homogenized samples were then centrifuged at 4 °C, 12,000 *g* for 20 min to separate cellular debris from the supernatant. The collected supernatant was quantified using a BCA (bicinchoninic acid) protein assay kit. Equal amounts of protein, based on the quantification results, were mixed with sample buffer (P0286, Beyotime) and heated at 90 °C for 10 min to denature. After cooling, the samples were subjected to electrophoresis, with the stacking gel run at 70 V for 30 min, followed by the separation gel at 120 V for 90 min. Protein transfer onto apolyvinylidene fluoride membrane was performed at 250 mA for 90 min. The membrane was blocked with tris-buffered saline with Tween-20 containing 5% nonfat milk for 1 h at room temperature, then incubated overnight at 4 °C with primary antibody including GFAP (1:500, 3670, CST), NF-L (1:1,000, 2837, CST), ChAT (1:500, 20747-1 -AP, Proteintech), GAPDH (1:50,000, 60004-1-Ig, Proteintech), and Alpha tubulin (1:20,000, 80762-1-RR, Proteintech), followed by incubation of horseradish peroxidase-conjugated secondary antibody (1:1,000, 7074S, CST) for 90 min at room temperature.

#### Immunofluorescence

Mouse brains embedded in OCT compound were sectioned at a thickness of 40 μm using a cryostat (Leica). The sections were rinsed with PBS and subsequently blocked at room temperature for 1 h in PBS containing 5% bovine serum albumin (BSA, ST023, Beyotime) and 0.5% Triton X-100. Primary antibodies including CREB (1:50, 9197, CST), pCREB (1:1,000, 9198, CST), c-Fos (1:1,000, 226 008, Synaptic Systems) and Iba1 (1:50, 17198, CST) were diluted in PBS containing 3% BSA and 0.3% Triton X-100, and the sections were incubated overnight at 4 °C. After incubation, the sections were rinsed 3 times with PBS and then incubated with the corresponding secondary antibodies including Alexa Flour 488 (1:500, A-11034, Thermo Fisher) and Cy3 IgG (1:500, GB21303, Servicebio), diluted in the same buffer, for 1.5 h at room temperature. Following 3 PBS rinses, sections were counterstained with 4′,6-diamidino-2-phenylindole (DAPI, G1012, Servicebio) for 10 min; after incubation, the sections were immediately rinsed 5 times with PBS and mounted for imaging. Photos were captured using *LAS X* (Leica) then analyzed using ImageJ (NIH).

#### Enzyme-linked immunosorbent assay

The ELISA kits including cAMP (CEA003Ge, Cloud-Clone), IL-1β (MEA563Mu, Cloud-Clone; EK201BHS, Multisciences), IL-10 (MEA056Mu, Cloud-Clone; EK110HS, Multisciences), TNF-α (MEA133Mu, Cloud-Clone; EK282HS/4, Multisciences), IL-6 (MEA079Mu, Cloud-Clone; EK206HS, Multisciences), and BDNF (MEA011Mu, Cloud-Clone; EK2127, Multisciences) were used to analyze brain samples. Frozen samples were thawed on ice and homogenized according to the manufacturer’s instructions, protein concentration was quantified using the BCA assay to ensure equal concentration across samples, and quantified samples were then measured according to the manufacturer’s protocol.

#### EEG recording

EEG recordings were performed using the 4-electrode system to monitor cortical activity in freely moving mice (in 30 cm × 30 cm × 30 cm transparent box) after binocular stimulation. Animals were anesthetized with tribromoethanol and placed in a stereotaxic apparatus for electrode implantation. Stainless-steel screw electrodes were implanted over the frontal and parietal cortices (anterior–posterior: +1.5 mm and –2.0 mm from bregma; mediolateral: ±1.5 mm), with 2 additional reference and ground electrodes positioned over the cerebellum. All electrodes were secured to the skull with P60 light-curing resin (3M), and animals were allowed to recover for 7 d before recording.

Continuous EEG signals were recorded for 24 h with a 12-h light/12-h dark cycle. The light and dark phases were analyzed separately to assess state-dependent neural activity. Signals were amplified, band-pass filtered between 0.5 and 100 Hz, and digitized at a sampling rate of 1 kHz. Artifacts related to movement were excluded prior to analysis. EEG data were processed using inserted software for power spectral analysis and sleep–wake state classification.

#### Open-field test

The OFT was conducted following standard protocols [10]. Mice were moved to the experimental environment 1 day before testing for acclimatization. The test was performed in a gray, nonreflective square open-field box (40 cm × 40 cm× 40 cm), with a 25 cm × 25 cm central area marked as central area. Mice were placed in a corner of the box and allowed to freely explore for 10 min. Their movement within the central area was recorded as an indicator of activity and anxiety-like behavior. Upon completion of the test, mice were returned to their home cages and the open-field box was cleaned with 75% ethanol.

#### Novel object recognition

The NOR test was conducted the day following the OFT, using the same test arena and lighting conditions (50 lux). During the initial training phase, 2 identical 50-ml white centrifuge tubes were placed at opposite diagonal corners of the arena. Mice were released into a nondiagonal corner and allowed to explore the environment for 10 min.

After an intertrial interval of 1.5 h, the NOR test was conducted. To prevent odor interference, the objects were replaced with new items: a 50-ml white centrifuge tube and a white T25 cell culture flask. Mice were allowed to explore the arena for 10 min, and the time spent sniffing each object was recorded during the first 20 s. The ratio of time spent sniffing the novel object to the total sniffing time was used as the final measure of the test outcome. Results will be shown as a recognition index:

$$\text{Recognition index}=\frac{\text{Time spent sniffing}\;T25\;\text{cell culture flask}}{\text{Total sniffing time}\;\left(20\;s\right)}$$ (3)

#### Morris water maze

The MWM task was conducted following standard protocols [10]. The water maze consisted of a circular pool (diameter: 1.2 m; depth: 30 cm) filled with water maintained at 18 to 20 °C and colored with edible carbon black dye for platform hiding. The experiment was divided into 2 phases: training and testing. During the training phase (days 1 to 5), mice were placed at 1 of 4 entry points located in quadrants opposite the submerged platform, which was positioned 1 cm below the water surface. Mice were allowed a maximum of 60 s to locate the platform; if failed, they were gently guided to it. Upon reaching the platform, they were permitted to rest for 15 s to consolidate spatial memory. Four trials were conducted each day, with the entry point randomized between trials. On day 6, the platform was removed, and mice were placed in the pool from the opposite platform area and allowed to swim for 60 s. The number of crossings over the platform’s previous location and the time spent in the target quadrant were recorded as measures of spatial memory. All ethological analyses were performed using Smart 3.0 (RWD).

#### Statistical analysis

Experiment data in this article were analyzed using GraphPad 10 (R&D) and R (Lucent Technologies), expressed as mean ± SEM. Unless specifically mentioned for comparisons between 2 groups, an unpaired 2-tailed Student *t* test was used. For comparisons among multiple groups, one-way analysis of variance (ANOVA) or two-way ANOVA with Tukey’s multiple comparison test were applied. A *P* value of <0.05 were considered statistically significant.

## Data Availability

All data necessary to understand and assess the conclusions of this study are available in the main text or the Supplementary Materials. There are no restrictions on data availability in the manuscript.
