# Supplementary material for: Noninvasive Terahertz Wave Binocular Stimulation Improves Cognition in Amyloid-β-Related Dementia
Source: Research (Wash D C). 2026 May 7;9:1268. doi: 10.34133/research.1268 (PMC13150075; doi:10.34133/research.1268)
Supplement: Supplementary 1 — Figs. S1 to S6 Table S1 [file research.1268.f1.docx]

**Supplementary Figure**


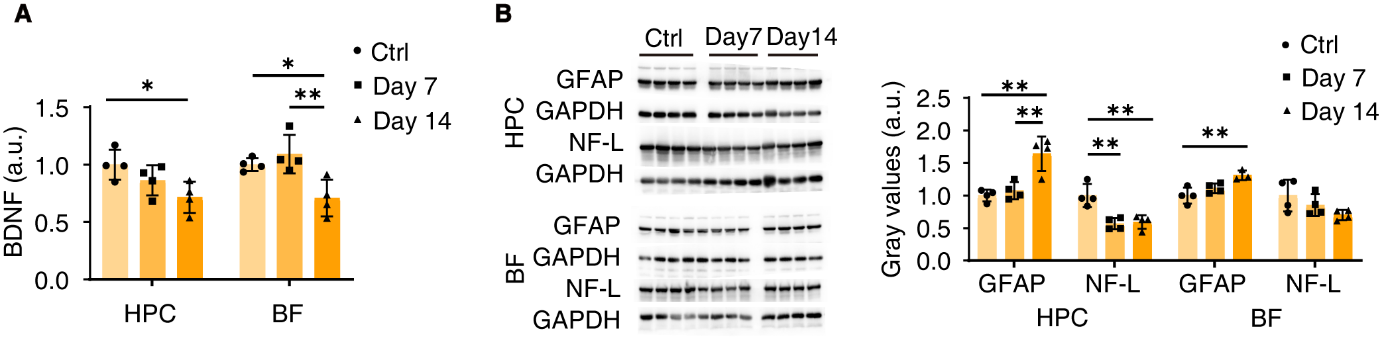


**Figure S1.** Detection of molecular biological indicators in Aβ_1-42_-induced dementia mice.

(A) Relative levels of BDNF in HPC and BF from mouse 7/14 days after Aβ_1-42_ injection measured by ELISA (n=4 per group, normalized to control). (B) Relative gray values of GFAP and NF-L in HPC and BF from mouse 7/14 days after Aβ_1-42_ injection measured by WB (n=4 per group, normalized to control). Data are shown as means ± SEM. **P*< 0.05, ***P*< 0.01; ns, not significant. One-way ANOVA was used for (A), (B).


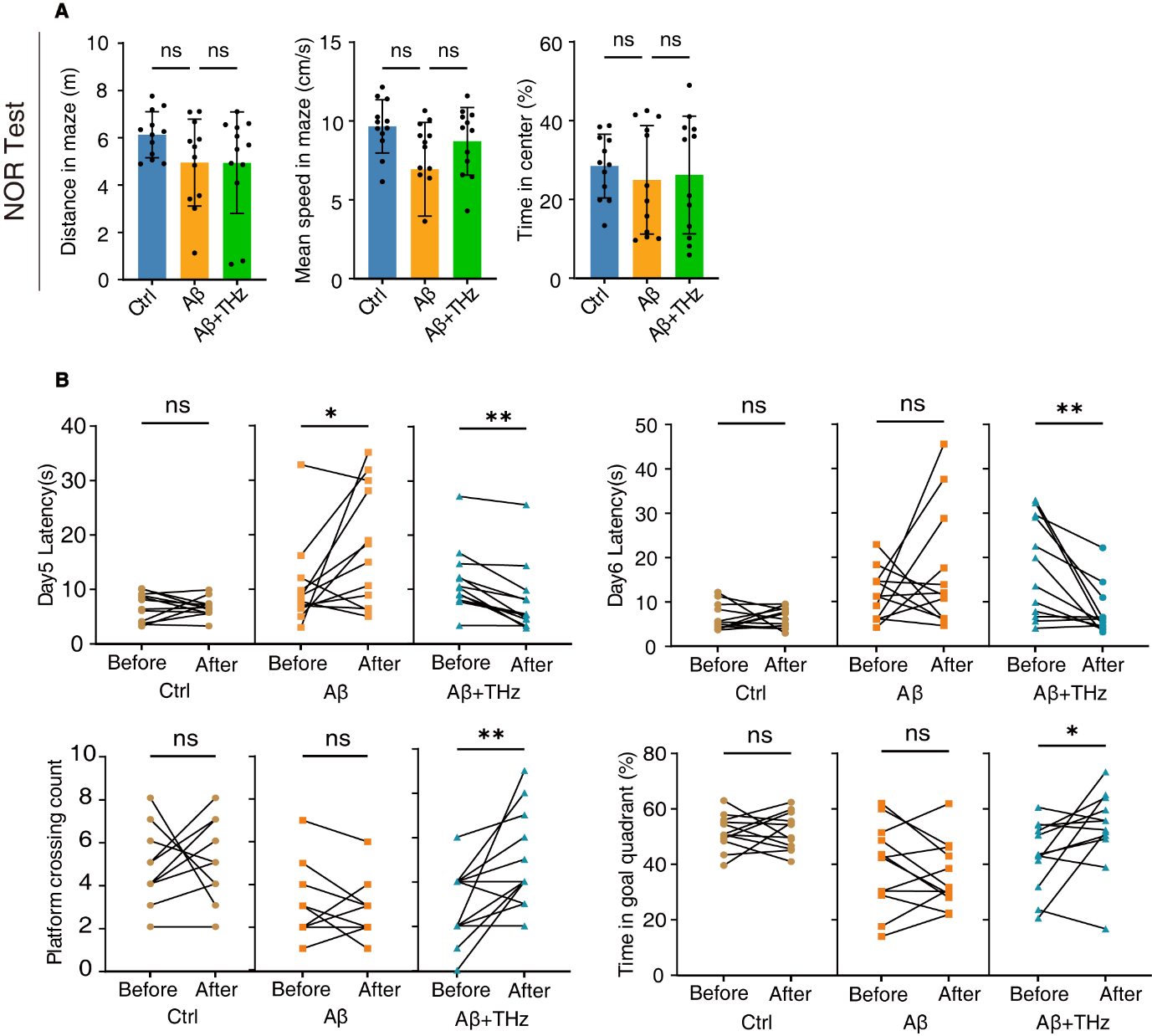


**Figure S2.** THz wave binocular stimulation enhances cognitive function in Aβ mice.

(A) Result of open filed test as follow: total distance (cm) traveled, mean speed (cm/s) and distance traveled in center area (n = 12 per group). (B) Result of escape latency (s) in the final training phase, escape latency (s) in testing phase, number of platforms crossing and time in goal quadrant, comparing mice before and after THz wave binocular stimulation in two steps of MWM tests (n=12 per group). Data are shown as means ± SEM. **P*< 0.05, ***P*< 0.01; ns, not significant. One-way ANOVA was used for (A); 2-tailed paired Student t test was used for (B).


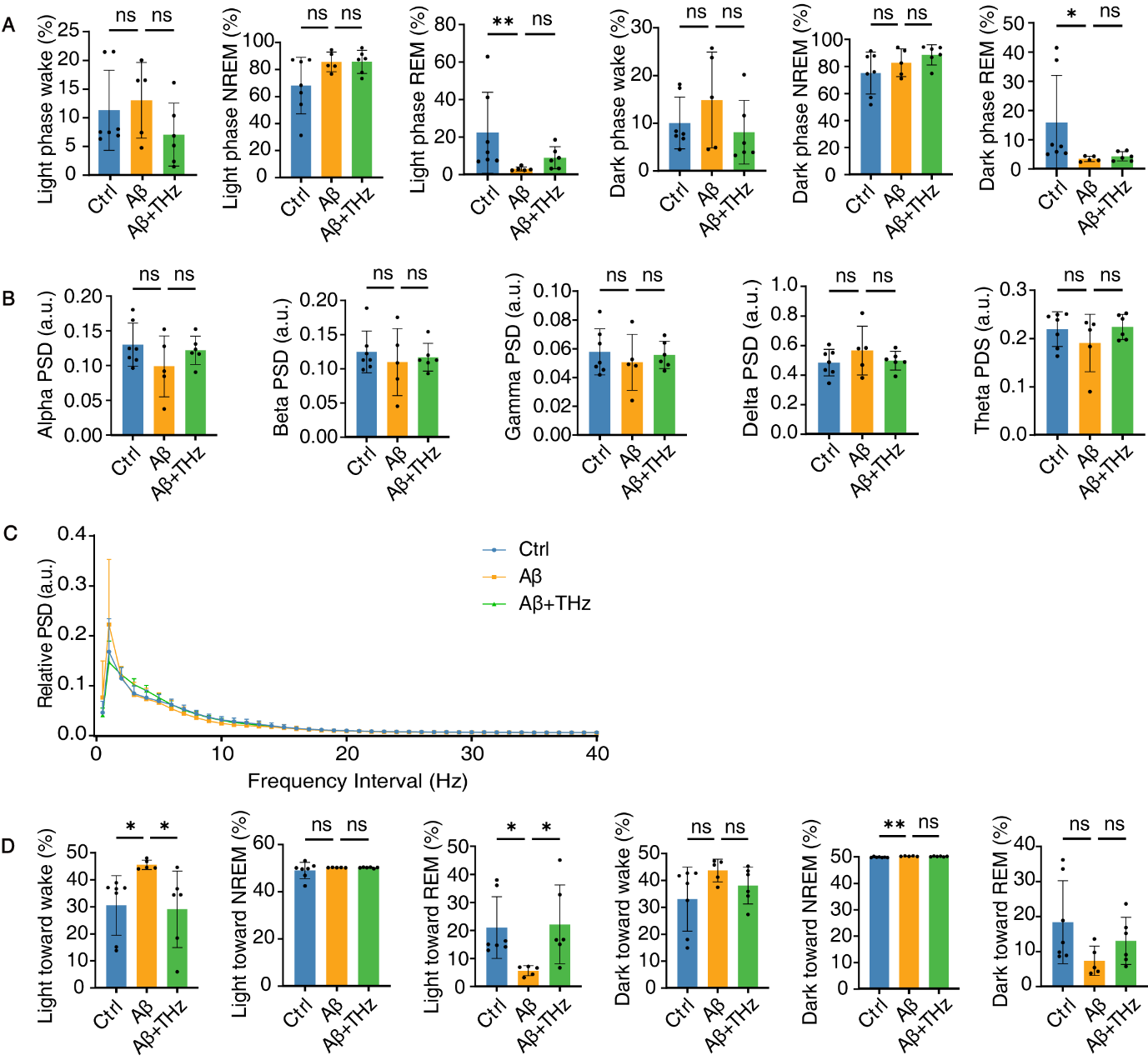


**Figure S3.** Changes in mice brain electrocardiograms following terahertz wave irradiation.

(A) Percentage of time spent in Wake, NREM, and REM states during light phase and dark phase. (B) Quantification of relative power in δ (0.5–4 Hz), θ (4–8 Hz), α (8–13 Hz), β (13–30 Hz), and γ (30–50 Hz) frequency bands. (C) Power spectrum density in the 0.5–50 Hz frequency range. (D) Proportion of transitions toward Wake, NREM, and REM states during light phase and dark phase. Data are shown as means ± SEM. **P*< 0.05, ***P*< 0.01; ns, not significant. Kruskal–Wallis test followed by Dunn’s multiple-comparison test were used for (A), (B), (D).

**
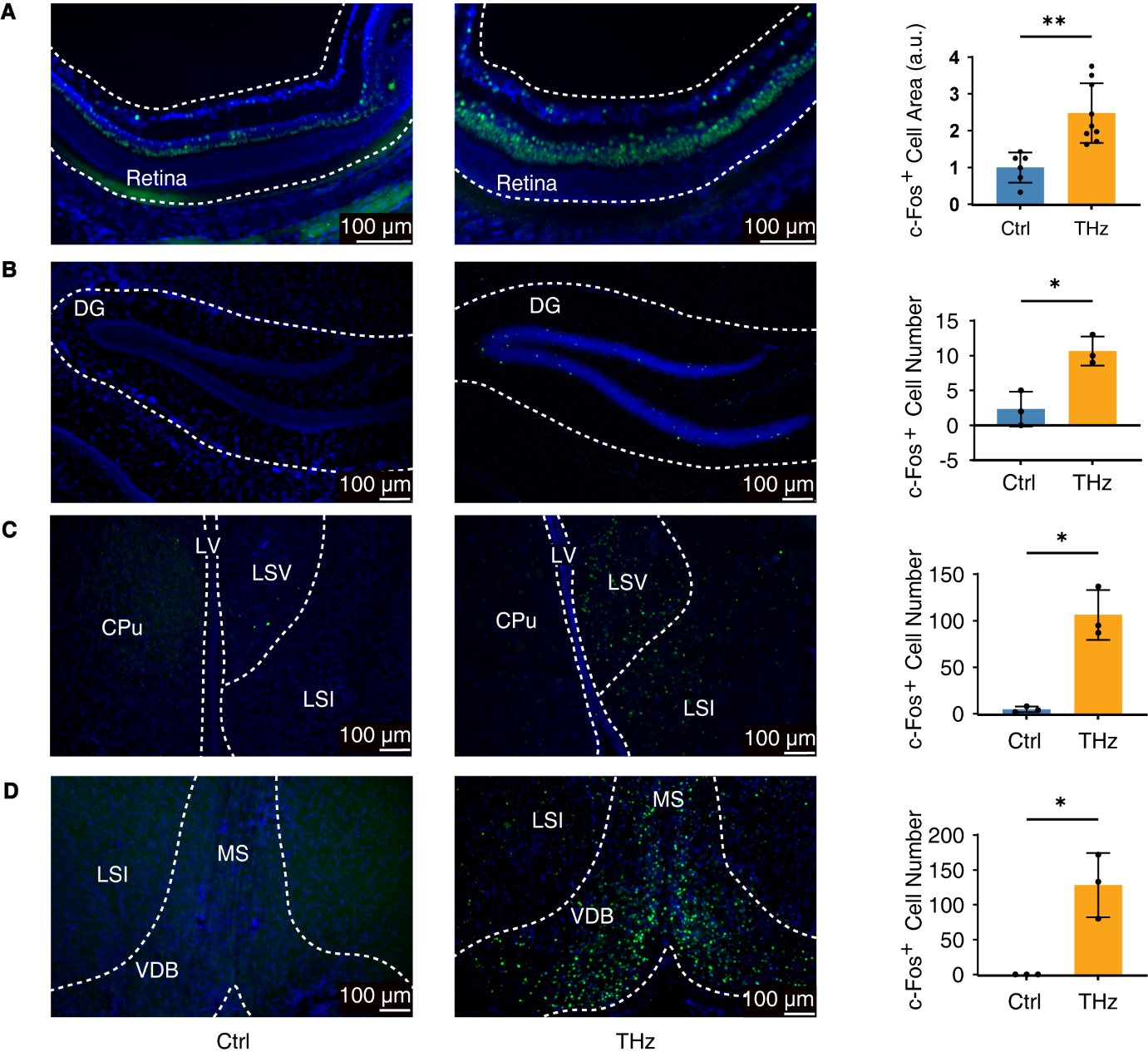
**

**Figure S4*.*** c-Fos immunofluorescence reveal influence of THz wave binocular stimulation toward brain.

(A) Immunofluorescence staining for c-Fos revealed an increase of neuronal activity in the retina following binocular stimulation (Scale bar: 100 μm, n=6-9 per group). (B) Immunofluorescence staining for c-Fos revealed an increase of neuronal activity in the hippocampal DG region following binocular stimulation (Scale bar: 100 μm, n=3 per group). (C) Immunofluorescence staining for c-Fos revealed an increase of neuronal activity in the ventral part of the lateral septal nucleus (LSV) following binocular stimulation (Scale bar: 100 μm, n=3 per group). (D) Immunofluorescence staining for c-Fos revealed an increase of neuronal activity in the vertical limb of the diagonal band nucleus (VDB) and medial septum (MS) following binocular stimulation (Scale bar: 100 μm, n=3 per group). Data are shown as means ± SEM.**P* < 0.05; ***P* < 0.01; ns, not significant. Statistical analysis was performed using the unpaired t-test with Welch’s correction for (A-D).


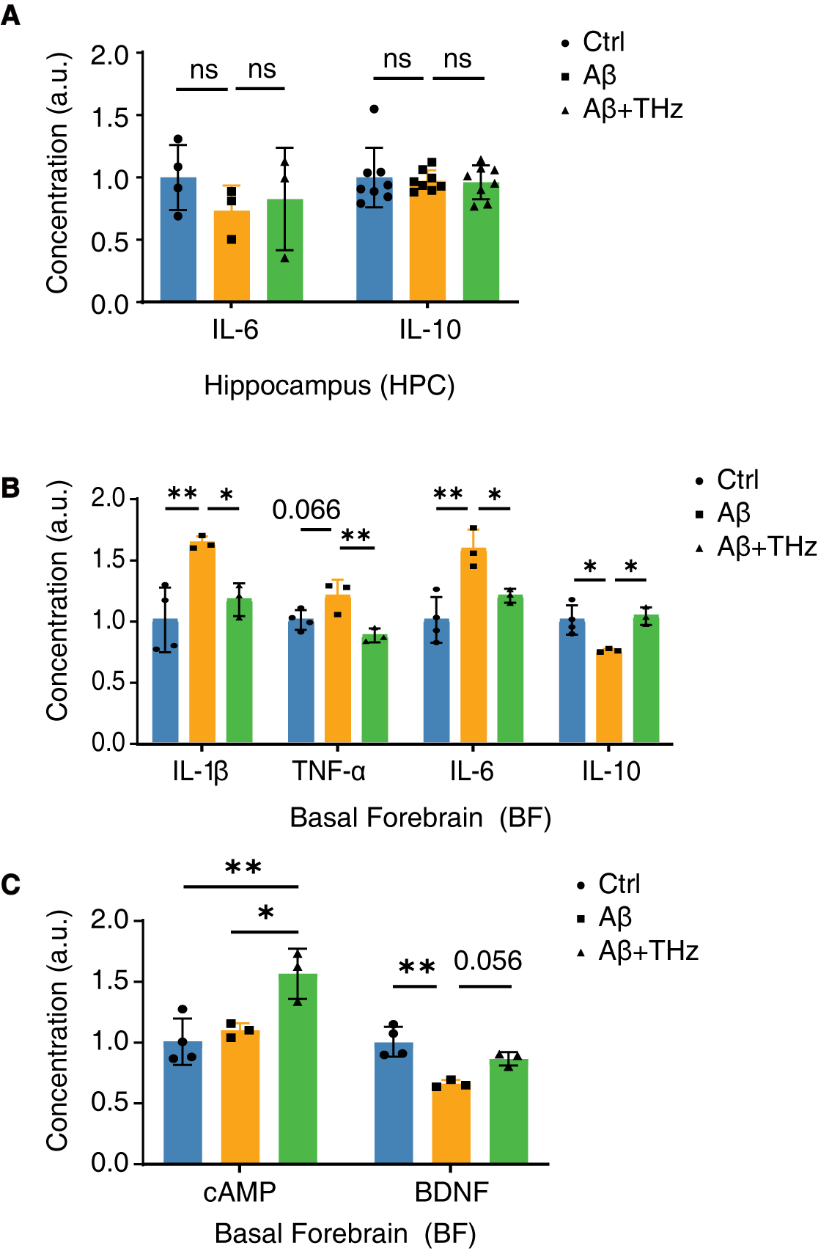


**Figure S5.** ELISA measurement of key biomarkers in HPC and BF.

(A) Relatively IL-6 and IL-10 levels in HPC of dementia mice with/without binocular stimulation for 2 weeks (normalized to control, n = 3-8 per group). (B) Relatively IL-1β TNF-α IL-6 and IL-10 levels in BF of dementia mice with/without binocular stimulation for 2 weeks (normalized to control, n = 3-4 per group). (C) Relatively cAMP and BDNF levels in BF in dementia mice with/without binocular stimulation for 2 weeks (normalized to control, n = 3-4 per group). Data are shown as means ± SEM. **P*< 0.05, ***P*< 0.01; ns, not significant. One-way ANOVA was used for (A-C).


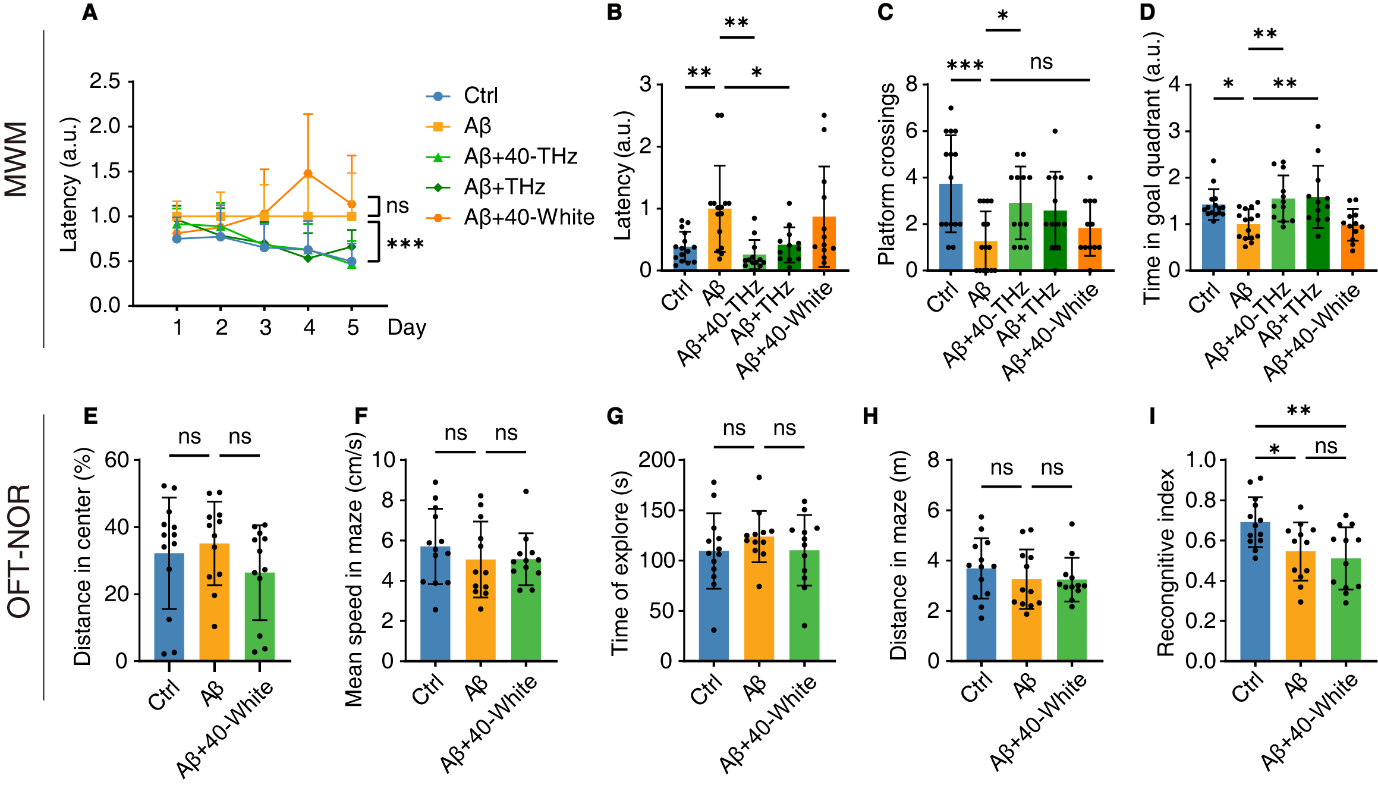


**Figure S6*.*** Differences in behavior test under different stimulation parameter.

(A) Progression of escape latencies (s) for the three groups across training days in the Morris water maze (n = 12-15 per group). (B) Escape latencies (s) during the probe trial (n = 12-15 per group). (C) Number of platforms crossing during the probe trial (n = 12-15 per group). (D) Time (s) spent swimming in the goal quadrant during the probe test (n = 12-15 per group). (E) Total distance (cm) traveled during open filed test (n = 12-13 per group). (F) Mean speed (cm/s) during open filed test (n = 12-13 per group). (G) Time spent (s) to explore both object in learning phrase (n = 12-13 per group). (H) Distance (m) traveled in center area during open filed test (n = 12-13 per group). (I) Recognition index of novel object recognition (NOR) test (n = 12-13 per group). Data are shown as means ± SEM. **P*< 0.05, ***P*< 0.01, ****P*< 0.001; ns, not significant. Two-way ANOVA with Tukey’s multiple comparison test was used for (A). One-way ANOVA was used for (B-I).

**Supplementary Table**

Tab. S1 Comparation between different non-pharmaceutical treatment.

| Article title | Stimulation | Method | Duration |
| --- | --- | --- | --- |
| Terahertz photons promote neuron growth and synapse formation through cAMP signaling pathway | 34.5 THz,  18 W/cm^2^ | optical fiber implantation,  anesthesia while treatment | 10 min/day for 3 days |
| Terahertz Wave Alleviates Comorbidity Anxiety in Pain by Reducing the Binding Capacity of Nanostructured Glutamate Molecules to GluA2 | 34 THz,  6 W/cm^2^ | optical fiber implantation,  anesthesia while treatment | 10 min/side for 2 side |
| Non-invasive, opsin-free mid-infrared modulation activates cortical neurons and accelerates associative learning | 56.3 THz,  100 W/cm^2^ | optical fiber implantation | 20 s/time 25 times/day for 5 days. |
| Sensory input-dependent gain modulation of the optokinetic nystagmus by mid-infrared stimulation in pigeons | 34.88 THz,  17 W/cm^2^ | optical fiber implantation,  anesthesia while treatment | 2 min/time real-time recording. |
| Terahertz Irradiation Improves Cognitive Impairments and Attenuates Alzheimer’s Neuropathology in the APPSWE/PS1DE9 Mouse: A Novel Therapeutic Intervention for Alzheimer’s Disease | 0.14 THz,  25 mW/cm^2^ | direct irradiation on head with skin removed,  anesthesia while treatment | 10 min/day, 5 days/week for 12 weeks. |
| Non-invasive modulation of meningeal lymphatics ameliorates ageing and Alzheimer’s disease-associated pathology and cognition in mice | 808 nm,  20 mW/cm^2^ | direct irradiation on head,  anesthesia while treatment | 10 min/time 3 times/week for 4 weeks |
| Terahertz Photons Improve Cognitive Functions in Posttraumatic Stress Disorder | 34.5 THz,  44 mW/cm^2^ | optical fiber implantation,  anesthesia while treatment | 10 min/side 2 side/day for 3 days. |
| 40 Hz light flickering facilitates the glymphatic flow via adenosine signaling in mice | white LED at 40 Hz | direct irradiation in cage | single 30 min |
| 40 Hz light flickering promotes sleep through cortical adenosine signaling | white LED at 40 Hz | direct irradiation in cage | single 30 min |
| Multisensory gamma stimulation promotes glymphatic clearance of amyloid | white LED and noise at 40 Hz | direct irradiation in cage | single 60 min |
| Multi-sensory Gamma Stimulation Ameliorates Alzheimer’s-Associated Pathology and Improves Cognition | white LED and noise at 40 Hz | direct irradiation in cage | 60 min/day for 15 days |
